# Comprehensive Profiling of T- and B-Cell Receptor Repertoires Demonstrates Impaired Developmental and Activation of Adaptive Immunity in DOCK8 Deficiency

**DOI:** 10.1007/s10875-026-02031-z

**Published:** 2026-05-06

**Authors:** Tal Beit-Halevi, Arnon Broides, Atar Lev, Amos J. Simon, Amit Nahum, Raz Somech, Yu Nee Lee

**Affiliations:** 1 Pediatric Department A and Immunology Service, Jeffrey Modell Foundation Center, Edmond and Lily Safra Children’s Hospital, Sheba Medical Center, Tel Hashomer, Israel; 2 School of Medicine, Faculty of Medical & Health Sciences, Tel Aviv University, Tel Aviv, Israel; 3 Pediatric Immunology Clinic, Joyce & Irving Goldman Medical School, Faculty of Health Sciences, Soroka University Medical Center, Ben Gurion University of the Negev, Beer Sheva, Israel; 4 Hemato-Immunology Unit, Hematology Lab, Sheba Cancer Research Center, Sheba Medical Center, Tel Hashomer, Israel; 5 Pediatrics Department A, Faculty of Health Sciences, Soroka University Medical Center, Ben Gurion University of the Negev, Beer Sheva, Israel; 6 Pediatric Department A and the Immunology Service, Edmond and Lily Safra Children’s Hospital, Sheba Medical Center, Tel Hashomer, 52621 Israel

**Keywords:** DOCK8 deficiency, Primary immunodeficiency, Inborn Errors of Immunity (IEI), T-cell receptor repertoire, B-cell receptor repertoire, Next generation sequencing

## Abstract

Dedicator of cytokinesis protein 8 (DOCK8) is a crucial regulator for the formation of immune synapses, allowing for a proper function of innate and adaptive immune systems. DOCK8 deficiency is a primary immunodeficiency, currently known as Inborn Errors of Immunity (IEI) affecting both cellular and humoral immunity, thus leading to various clinical presentations ranging from infections, autoimmunity, inflammations and malignancies. We aimed to identify unique molecular signatures in the adaptive immune repertoire of DOCK8-deficient patients through comprehensive sequencing and analysis of the T-cell receptor (TCR) and B-cell receptor (BCR) repertoires, correlating these signatures with clinical features of the patients, ultimately providing a “fingerprint” of both individual’s immunological status and disease conditions. We characterized the TCR repertoires of αβ T- and γδ T-cells, together with BCR repertoires of B-cells, in peripheral blood of 13 patients and age matched healthy donor controls using next generation sequencing. TCR repertoire in patients with DOCK8 deficiency demonstrated restricted diversity and clonal expansions, favorable use of specific gene in the *V-D-J* recombination process, and a unique finding of longer complementarity-determining-region-3 (CDR3) length, revealing an impaired T-cell development and activation. Furthermore, BCR repertoire demonstrated high diversity without clonal expansions, portraying an abnormal B-cell development leading to a possible mechanism for atopy and autoimmunity, alongside inadequate activation in DOCK8 deficiency. Lastly, various *DOCK8* mutations demonstrated a more profound defects in immune repertoire. These findings expand our knowledge on the role DOCK8 plays in shaping the TCR and BCR repertoires in DOCK8 deficiency.

**Supplementary Information:**

The online version contains supplementary material available at 10.1007/s10875-026-02031-z.

## Introduction

Dedicator of cytokinesis 8 (DOCK8) deficiency, also known as DOCK8 immunodeficiency syndrome (DIDS), is a combined type of Inborn Errors of Immunity (IEI) hence affecting both cellular and humoral immunity. The *DOCK8* gene is located on chromosome 9, spanning over 250 kilobases, encoding a large protein highly expressed in hematopoietic tissues [1–3]. The DOCK8 protein functions as an atypical guanine nucleotide exchange factor (GEF), which leads to actin polymerization and cytoskeletal rearrangement, necessary processes for the formation of immunological synapses and immune cell migration [1, 4, 5]. Thus, loss of DOCK8 leads to actinopathy, profoundly impacting both innate and adaptive immunity, including dendritic cell and lymphocyte migration and functions, memory responses of T- and B-cells and antibody production [4–9]. Recent studies have highlighted the specific roles of regulatory T-cells [10–12] and neutrophils [13] in eczema, as well as the developmental inhibition of B-cell subsets and impaired B-cell receptor signaling [14] associated with DOCK8 deficiency. Furthermore, the *DOCK8* locus is prone for large germline deletions as well as recombination-mediated somatic DNA repair due to repetitive sequences [15–19]. Somatic reversions partially restore DOCK8 expression in some cell lineages, predominantly within T-cells, influencing the genotype-phenotype relationship [17, 20]. Specifically, loss-of-function deletions mutations in *DOCK8* are found mainly in consanguineous populations, where the possibility of somatic repair is low thereby resulting in a severe disease [4, 15, 16, 18, 19, 21–23].

DOCK8 deficiency is characterized by wide-ranging clinical sequelae, including atopy, recurrent infections with susceptibility to cutaneous viral infections, autoimmunity and malignancy [4, 16, 19, 22, 24, 25]. As initially described as an autosomal recessive hyper immunoglobulin (Ig) E syndrome (AR-HIES), the laboratory profile of DOCK8 deficiency typically include elevated IgE, alongside normal to elevated IgG and IgA, low IgM, eosinophilia, and lymphopenia with occasionally low number of T-cell receptor excision circles (TRECs) [15, 21, 23, 26, 27]. Generally, DOCK8 deficiency presents during early years of life, where about 80% have life-threatening complications and about 50% succumb to death by the age of 20 years [24]. Thus, early hematopoietic stem cell transplantation (HSCT) is strongly advised as a potential curative treatment in light of the high morbidity and mortality associated with DOCK8 deficiency [28].

T- and B-cells in the adaptive immune system use diverse T-cell receptors (TCRs) and B-cell receptors (BCRs) to recognize unlimited number of different foreign antigens and to neutralize them. TCRs, expressed either by the αβ T-cells (*TRA* and *TRB* genes, respectively) or γδ T-cells (*TRG* and *TRD* genes, respectively), and BCRs, composed of heavy and light chains (*IGH* and *IGL*, respectively), exhibit a variable region crucial for antigen recognition. Thus *TRA* and *TRB* repertoires represent αβ T-cells, *TRG* and *TRD* repertoires represent γδ T-cells and *IGH* and *IGL* repertoires represent B-cells. The TCR and BCR repertoire diversity of the variable region is achieved through the *V(D)J* recombination process, which is the combinatorial joining of numerous *V-D-J* (variable, diversity, and joining) gene segments. Further diversification occurs via imprecise joining of gene segments during the process of *V(D)J* recombination, somatic hypermutation in B-cells, and combination of different chains [29, 30]. Understanding the T- and B-cell diversity is of a great importance, and next generation sequencing (NGS) technologies allow simultaneous sequencing of millions of sequences, enabling a detailed understanding of the adaptive immune system at a molecular level. NGS-based immune repertoire studies on various Inborn Errors of Immunity (IEI) have been conducted previously [31–33], including BCR immune repertoire characterization in 3 patients with DOCK8 deficiency [34] and IEIs with similar pathomechanism of actin cytoskeletal regulation [4, 35, 36], such as Wiskott-Aldrich syndrome (WAS) [37–39] and *ARPC1B* deficiency [40, 41]. Recently, a unique TCR repertoire of CD8 + was characterized in DOCK8 deficient patients by a comprehensive analyses of αβ T-cell receptor repertoire [42]. However, a comprehensive characterization of αβ and γδ T-cell receptor repertoire, together with B-cell receptor repertoire for each patient with DOCK8 deficiency using NGS has not been done yet.

As DOCK8 deficiency affects both T- and B-cells, we aimed to characterize both TCR and BCR repertoires in peripheral blood of patient with DOCK8 deficiency using NGS, by analyzing the sequencing data of *TRB* repertoire for αβ T-cell, *TRG* repertoire for γδ T-cell and *IGH* repertoire for B cells. We aimed to uncover correlations between immune repertoire findings, *DOCK8* mutations, and clinical and laboratory data of DOCK8 deficient patients, while defining a specific molecular signature for DOCK8 deficiency with distinctive features in comparison to other IEIs with similar pathomechanism.

## Methods

### Patients

Patients with DOCK8 deficiency, also known as DOCK8 immunodeficiency syndrome (DIDS), are children under the age of 18 years, who were diagnosed at the “Edmond and Lily Safra” Children’s Hospital, Sheba Medical Center at Tel HaShomer, Israel. The patient group consists of 13 patients with DIDS diagnosis according to clinical manifestations and genetic sequencing. The control group consists of 5 healthy children, under the age 18, were included to cover the age range of the patients (5 months – 17 years old), with no clinical and immunological abnormalities. Our control samples are from admitted pediatric patients confirmed to be eligible to be used as controls. A written informed consent was given by the parents of all patients and healthy controls. The study was approved by the IRB of the Sheba Medical Center, Ramat Gan, Israel and was conducted in accordance with the Declaration of Helsinki.

### Clinical Description and Laboratory Data Including Immunological Evaluations

Clinical and laboratory data were obtained from the electronic medical record system of the Sheba Medical Center. For immunological evaluations, cell surface markers of peripheral blood mononuclear cells (PBMCs), quantitation of TCR Variable β (Vβ) repertoire in PBMCs, lymphocyte proliferative responses to mitogens, and the amount of signal joint TRECs are determined as previously described [43]. Serum concentration of immunoglobulins was measured by nephelometry.

### Genetic Evaluation

Genomic DNA was isolated from peripheral blood samples of patients with DIDS and family members, and primers across the DOCK8 gene locus were directly sequenced by dideoxy Sanger sequencing. Resulting sequences were evaluated using Sequencer v5.0 software (Gene Codes Corporation).

### Immune Repertoire Determination By NGS

*TRB*, *TRG* and *IGH* repertoire libraries were generated from 150 ng of genomic DNA extracted from the patients’ and healthy controls’ peripheral blood using primers for conserved regions of *V* and *J* genes, according to manufacturer’s protocol (LymphoTrack, Invivoscribe Technologies, France and USA), amplified by multiplex polymerase chain reaction (PCR). Genomic DNA was extracted from whole blood without lymphocyte enrichment. *V(D)J* amplification targets rearranged alleles present only in lymphocytes, whereas in non-lymphoid blood cells, germline configurations place primers tens to hundreds of kilobases apart, preventing efficient PCR amplification. Furthermore, at the time of sampling the patients did not undergo active treatments. The resulting libraries were purified using AMPure XP beads (Beckman Coulter, USA) and were quantified using highly sensitive Qubit DNA quantification assays (Qubit, Thermo Fisher Scientific, USA). Furthermore, the libraries were subjected for quality control using the TapeStation system (DNA ScreenTape Analysis, Agilent Technologies, USA). Next, the libraries were pooled and sequenced using Mi-Seq Illumnia technology (Illumina Inc, USA).

The sequences were subjected to initial bioinformatic analyses using the software provided by Invivoscribe Technologies to generate FASTA sequence files for all the libraries, which then were submitted to the IMGT HighV-QUEST webserver (http://www.imgt.org), filtered for productive rearrangements and analyzed further for Hierarchical Treemap (Macrofocus Gmbh, Switzerland), Shannon’s H, and Simpson’s D diversity indices, sum of top clones, frequency of the different *V*,* D* and *J* gene usages and average CDR3 length. Shannon’s H and Simpson’s D were calculated using the following equations:

$$Shannon\backprime s\;H=-{\sum\:}_{i=1}^R\:p_i\:\:\mathrm{I}\mathrm{n}\:\:p_i$$

$$Simpson\backprime s\;D=\sum\:_{i=1}^R\:\:p_i^2$$

R = Total sequences; i = Unique sequences; pi = proportion of the total sequences belonging to the “i” ^th^ unique sequence.

CDR3 lengths for the *TRB* repertoire were determined using IMGT/HighV-QUEST, which defines CDR-IMGT as the region between the conserved cysteine (position 104) of the *V* gene segment and the conserved phenylalanine or tryptophan (position 118) of the *J* gene segment, thereby excluding these two anchor residues. Consequently, the reported CDR3 amino acid lengths in this study are two residues shorter than those typically obtained with tools that include the conserved amino acids in the CDR3 sequence [44].

All sequencing data generated in this study have been deposited in Figshare and are publicly available at 10.6084/m9.figshare.31996116.

### Generation of Immune Repertoire Database for DIDS Patients

TRB, TRG and IGH immune repertoire database for DIDS patients was generated using Data Explorer, Azure, Microsoft. This facilitated the analyses process.

### Statistical Analysis

Statistical analyses for unpaired Mann-Whitney U-test were carried out using the Prism11 (GraphPad Software Inc., USA). Multiple Mann-Whitney U-test with Benjammini, Krieger and Yekutieli method was applied to all families of comparisons to correct for multiple comparisons (i.e. *V*- gene usage, *D*-gene usage and *J*-gene usage). Spearman rank correlation between developmental and functional parameters and repertoire metrics were performed within the patient cohort only.

## Results

### Clinical, Genetic, and Immunological Evaluations of our DOCK8 Deficient Patients

Thirteen patient with DOCK8 deficiency who were previously described [45, 46] were included in our current study, with twelve having homozygous mutations and one a compound heterozygous mutation (Table 1). The patients were from consanguineous families (Middle Eastern descent), except for one from a non-consanguineous family. They were grouped based on mutation types: nonsense (Group 1), splicing (Group II), frameshift/deletion (Group III), and compound heterozygous (Groups IV) mutations (Table 1). Clinically, patients presented with eczema, allergies, asthma and recurrent viral, bacterial, and fungal infections, often complicated by bronchiectasis, and autoimmunity. Furthermore, DOCK8-deficient patients presented with classic manifestation of IEI such as failure to thrive (FTT), chronic diarrhea and hepatosplenomegaly (HSM). However, none had thrombocytopenia, a feature of other IEIs associated with WAS [21] and ARPC1B deficiency [47] with similar pathomechanism as DOCK8 deficiency (Table 1). In addition, eosinophilia, a prominent laboratory feature of DOCK8 deficiency [48], was prominent in all patients (Table 1).

**Table 1:** Demographic, genetic, clinical and laboratory data of patients with DOCK8 deficiency

Group	Group I						Group II			Group III			Group IV
Patients	P1 ¹	P2	P3 ¹	P4	P5	P6	P7	P8	P9	P10 ²	P11 ²	P12	P13
Mutation type	Homozygous; Nonsense						Homozygous; Splicing			Homozygous; Frameshift		Homozygous, Deletion	Compound Heterozygous; Missense Nonsense
Mutation	c.5134 C > A; p.S1711X						c.IVS5 + 1G > C			c.5415-5424del insATCCTGTTCTTTGTA; p.D1806SfsX5		Ex42_ 45del	c.1418 A > G; p.K473R c.571 A > T; p.K191X
Exon	40						5			42		42–45	12 and 5
Age at diagnosis	7.5 y	Dx after death	4.5 y	5 mo ^a^	17 y	2 y + 9 mo	2 y	3.5 y	NA	5 y	2 y ^a^	8 y	11.5 y
Clinical manifestation													
Atopy	Eczema; Milk allergy	Eczema	Eczema; Asthma	Eczema; Asthma; Food allergy	Eczema; Asthma	Eczema	Eczema	Eczema; Food allergies	NA	Eczema	Eczema	Asthma	Eczema; Food allergies
Recurrent infections	AOM; Herpes stomatitis; Pneumonia	AOM; Pneumonia; Mucocutaneous Herpes; Herpes keratitis; Streptococcus, Pseudomonas and *E. coli* bacteremia/ sepsis	Giardia; Crypto-sporidium	Pneumonia	*Strepto-coccus* *pneumoniae*; *Verruca plana*	PJP	AOM; Pneumonia and lung abscesses; Muco-cutaneous infections (Impetigo, VZV, HSV)	AOM and AOE; Pneumonia; Muco-cutaneous infections (Impetigo, VZV, Candida)	NA	Pneumonia; Muco-cutaneous infections (Impetigo, HSV, scabies, candida); Crypto-sporidium	Sino-pulmonary infections; Skin infections (abscesses, HSV); Crypto-sporidium; CMV and EBV virmia; BCGitis	Pneumonia; Skin infections (HPV, Tinea corporis and capitis, Impetigo)	AOM; Pneumonia; Skin infections (HPV, HSV, Impetigo, Molluscum contagiosum, VZV)
Auto-immunity & auto-inflammatory	ITP		AIHA; Susp. AI hepatitis		Celiac		PSC; Arthritis and arthralgia			PSC	PSC	Aortic vasculitis	
Other	FTT	FTT; HSM	FTT; Diarrhea with PLE; Chronic lung disease	Blue sclera	FTT; End-stage lung disease	FTT; Dysentery; Anemia; Elevated liver enzymes	Bron-chiectasis; Chronic diarrhea; HSM; Anemia; Dilated aorta	Bron-chiectasis; Anemia; Splenomegaly	NA	FTT; Short stature; Bronchiectasis; Diarrhea; Anemia; Hepatomegaly	FTT; Short stature; HSM; Renal tubular disorder		Arthralgia; DDH; Drug allergy
CBC													
WBC (10³/µl)	NA	12.15	NA	NA	NA	NA	**↑ 21.94**	6.91	NA	**↑ 27.83**	**↑ 16.86**	7.36	**↑ 21.16**
AEC (10³/µl) ^b^	**↑ 3.40**	**↑ 11.07**	**↑ 30.00**	**↑ 0.56**	**↑ 2.20**	**↑ 29.27**	**↑ 5.16**	**↑ 1.49**	NA	**↑ 10.01**	**↑ 6.22**	**↑ 2.43**	**↑ 2.00**
Lymphocytes subsets, cells/µl ×10³													
Lymphocytes	NA	NA	NA	NA	NA	NA	5.39	2.47	2.05	**↑ 13.36**	ND	ND	**↓ 1.88**
CD3 + T cells	**↓ 0.60**	2.70	**↓ 0.72**	**↓ 1.43**	**↓ 0.47**	1.51	3.29	1.70	1.41	**↑ 8.42**	ND	ND	1.33
CD4 + T helper	**↓ 0.26**	**↓ 1.69**	**↓ 0.36**	**↓ 1.33**	**↓ 0.18**	0.88	**↓ 0.59**	**↓ 0.32**	**↓ 0.37**	2.00	ND	ND	0.83
CD8 + T cytotoxic	**↓ 0.34**	0.71	**↓ 0.31**	**↓ 0.09**	**↓ 0.26**	**↓ 0.38**	**↑ 2.21**	1.11	**↑ 0.96**	**↑ 4.54**	ND	ND	0.45
CD4/CD8 ratio	**↓ 0.76**	2.38	**↓ 1.16**	**↑ 14.78**	**↓ 0.69**	2.32	**↓ 0.27**	**↓ 0.29**	**↓ 0.38**	**↓ 0.44**	ND	ND	1.83
CD19+/20 + B cells	0.30	**↑ 4.31**	0.73	2.12	0.41	1.07	0.86	0.57	**↑ 0.51**	**↑ 3.87**	ND	ND	0.34
CD56 + NK cells	**↑ 0.90**	0.26	0.23	**↓ 0.12**	**↓ 0.05**	0.22	ND	ND	ND	ND	ND	ND	ND
CD56 + NK cells (%)	ND	ND	ND	ND	ND	ND	14	**↓ 3**	13	**↑ 18**	ND	ND	8
T cell function													
TREC (> 400)	ND	**↓ 190**	ND	**↓ 268**	**↓ 13**	**↓ 53**	ND	**↓ 243**	**↓ 31**	**↓ 110**	ND	**↓ 60**	790
TCR-Vβ	ND	Restricted/ No clonality	ND	Normal/ Polyclonal	ND	Oligoclonal	Oligoclonal	Restricted/ Skewed	Restricted/Clonal	Oligoclonal	ND	Oligoclonal	Normal/ Polyclonal
PHA 25 µg/ml proliferation (cpm) ^c^	**79%**	124%	**2%**	ND	**3%**	**86%**	133%	109%	ND	ND	ND	ND	ND
Anti-CD3 proliferation (cpm) ^c^	ND	**17%**	ND	ND	ND	ND	**94%**	**66%**	ND	ND	ND	ND	ND
Serum immunoglobulins levels ᵇ													
IgG (mg/dl) ^b, d^	**↑ 1**,**767**	**↑ 1**,**170**	1,260	613	973	1,119	**↑ 2**,**510**	**↑ 2**,**960**	**↑ 1**,**800**	**↑ 1**,**620**	**↑ 1**,**670**	1,400	**↑ 2**,**570**
IgM (mg/dl) ^b^	168	167	120	**↑ 429**	103	27	**↓ 40**	89	**↓ 29**	**↓ 55**	**↓ 46**	53	155
IgA (mg/dl) ^b^	**↑ 978**	74	**↑ 5**,**900**	88	**↑ 1**,**528**	**↑ 493**	**↑ 2**,**090**	**↑ 483**	191	163	**↑ 318**	229	**↑ 353**
IgE (IU/ml) ^b^	**↑ 3**,**350**	**↑ 135**,**000**	**↑ 1**,**388**	52	**↑ 1**,**122**	**↑ 282**	**↑ 4**,**430**	**↑ 17**,**900**	**↑ 3**,**820**	**↑ 16**,**500**	**↑ 5**,**720**	**↑ 3**,**610**	**↑ 7**,**651**
Specific antibodies	ND	* Positive: EBV; CMV; VZV; HSV * Negative: Measles; Mumps; Rubella; Polio	* Positive: Myco-plasma	* Negative: HBV	* Positive: EBV * Negative: Pneumo-coccus (post vaccines); Measles; Mumps; VZV; CMV	* Negative: HBV; Measles; VZV	* Positive: Rubella; Polio; VZV; EBV; CMV; HSV * Negtive: Measles; Mumps; HBV	* Positive: HBV; EBV; CMV; VZV; HSV	ND	* Positive: HBV; Polio; Rubella; HSV; CMV; VZV; EBV * Negative: Measles; Mumps	* Positive: HBV	* Positive: Rubella; Polio; VZV; CMV; HSV * Negative: Measles; Mumps	* Positive: Mumps; Rubella; VZV; HSV; EBV * Negative: Measles; HAV; HBV; Tetanus, Diphtheria; CMV; Toxoplasma gondii.
References	These patients were previously reported (37), and additional data was added from electronic medical record system of the Sheba Medical Center						These patients were previously reported (38), and additional data was added from electronic medical record system of the Sheba Medical Center						

Age-matched reference ranges/control are based on published data (41, 42) and electronic medical record system of the Sheba Medical Center. Abnormal values are bolded and arrows indicate whether higher or lower than the reference values.

*AEC* absolute eosinophil count, *AI* autoimmune, *AIHA* autoimmune hemolytic anemia, *AOE* acute otitis externa, *AOM* acute otitis media, *BCG* bacille Calmette-Guerin, *CD* cluster of differentiation, *CMV* cytomegalovirus, *Dx* diagnosis, *EBV* Epstein-Barr virus, *FTT* failure to thrive, *HAV* hepatitis A virus, *HBV* hepatitis B virus, *HPV* human papillomavirus, *HSM* hepatosplenomegaly, *HSV* herpes simplex virus, *Hx* history, *Ig* immunoglobulin, *ITP* immune thrombocytopenia purpura, *mo* months, *NA* not available/applicable, *ND* not done, *NK* natural killer, *PHA* phytohemagglutinin, *PJP* pneumocystis jiroveci pneumonia, *PLE* protein-losing enteropathy, *Pt.* patient, *Rec.* recurrent, *Susp.* suspected, *TCR-Vβ* T cell receptor Variable β, *TREC* T-cell receptor excision circles, *VZV* varicella zoster virus, *WBC* white blood cells, *y* years

^1,2^ Siblings

^a^ Diagnosis due to family history

^b^ Highest value evaluated during disease course and before hematopoietic stem cell transplant (if done)

^c^ [³H]Thymidine uptake in response to mitogens, percent of control

^d^ IgG concentrations before intravenous immunoglobulin (IVIG) therapy

Immunological evaluation showed that half of the patients had decreased CD3^+^ T-cells, and most had abnormal CD4^+^/CD8^+^ ratios and majority of patients had within the normal range CD19^+^/CD20^+^ B-cells, except three patients that had elevated levels (Table 1) [49, 50]. Furthermore, T-cell function was compromised with reduced thymic output (TCR excision circles - TREC), restricted TCR diversity (TCR Variable β - TCR-Vβ) and generally reduced T-cell responses to mitogenic stimulation using phytohemagglutinin and anti-CD3 (Table 1). Serum immunoglobulin levels varied, with some patients having protective antibody levels against various pathogens. These clinical and immunological evaluations further emphasize the combined nature of immunodeficiency of both T- and B-cell types associated with DOCK8 deficiency.

### TCR and BCR Repertoire Sequencing from Peripheral Blood of Patients with DOCK8 Deficiency using NGS

As DOCK8 deficiency affects both T- and B-cell immunity, we used NGS for an in-depth sequencing of the variable regions in the *TRB*, *TRG* and *IGH* loci of 13 patients and 5 healthy controls. The average age at sampling was 4.75 and 3.17 years for DOCK8 deficient patients’ group and controls’ group, respectively, (Table S1), which showed no significance differences (Figure S1). We obtained on an average 84,593 and 57,360 total sequences for T-cells in patients with DOCK8 deficiency and healthy controls, respectively. Furthermore, we obtained on an average 220,467 and 113,171 total sequences for B-cells in patients with DOCK8 deficiency, and healthy controls, respectively. The specific number of sequences for each individual is specified in Table S1.

### The *TRB* and *TRG* Repertoires are Restricted but *IGH* Repertoire is Diverse in the Peripheral T- and B- Cells of Patients with DOCK8 Deficiency

**Fig. 1:**
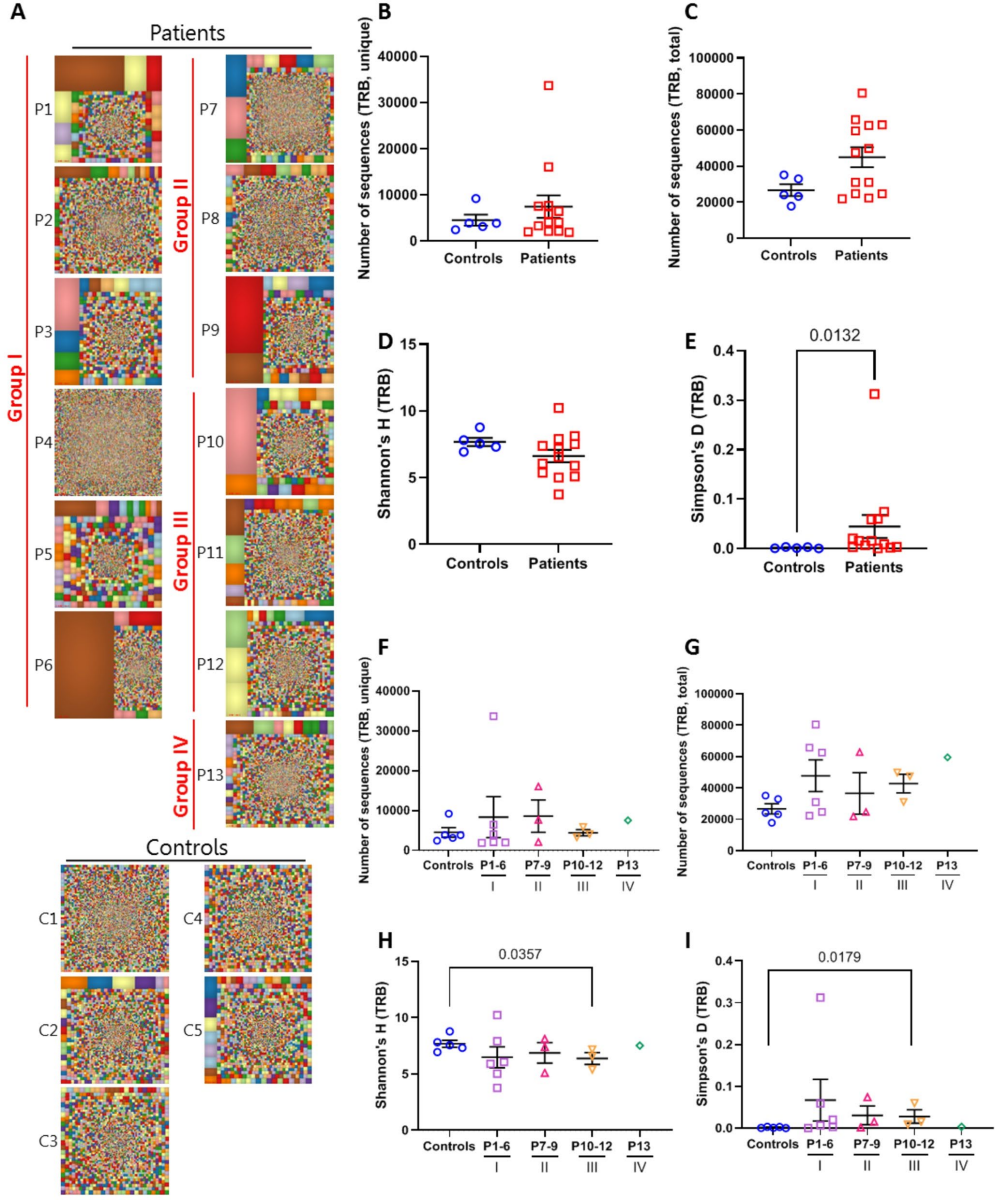
The T cell receptor β (*TRB*) repertoire diversity in DOCK8 deficiency. (**A**) Treemap illustrations graphically and qualitatively present the diversity in *TRB* repertoire for patients and controls, where each square represents a specific clone and the size of the square represent the frequency of the clone. Scatter dot plots for patients and controls presenting: (**B**) The unique number of sequences, which reflects the number of different clonotypes; (**C**) The number of total sequences, which portray the number of all circulating cells; (**D**) Shannon’s H index for diversity; and **(E)** Simpson’s D index of unevenness. Scatter dot plots comparing between controls and different groups of patients based on DOCK8 mutation type: (**F**) The unique number of sequences; (**G**) The number of total sequences; (**H**) Shannon’s H index for diversity; and **(I)** Simpson’s D index of unevenness. The whiskers in the graphs (**B-I**) present standard error (± SE). Statistics (**B-I**) performed using the Mann-Whitney U-test

To study the circulating αβ T-cell population, we analyzed the *TRB* repertoire. Hierarchical Treemaps were used to graphically present the overall repertoire diversity, where each square represents a different recombination, and the square’s size represents its frequency relative to all the sequences obtained. Overall, the *TRB* repertoire of majority of DOCK8 deficient patients was restricted and clonally expanded compared to controls (Fig. 1A). Next, we analyzed the number of sequences of the *TRB* repertoire. The unique number of sequences reflects the number of different clonotypes, and the number of total sequences portray the number of circulating αβT-cells. No significant finding was observed regarding the number of unique (Fig. 1B), and total (Fig. 1C) sequences in patients with DOCK8 deficiency compared to controls. The diversity of *TRB* repertoire was slightly lower in DOCK8 deficient patients compared to controls, as demonstrated by a lower Shannon’s H index (Fig. 1D). The Simpson’s D index of unevenness for the TRB repertoire of the patients was significantly higher compared to controls (Fig. 1E), indicative for an increased uneven repertoire and clonal expansion. Furthermore, we wanted to determine whether these repertoire diversity measures were affected differently based on the different *DOCK8* mutations of our patients (Table 1; Fig. 1, F-I). Only group III showed a significantly lower Shannon’s H and higher Simpson’s D index (Fig. 1, H and I). These analyses demonstrate that DOCK8 deficiency reduces *TRB* repertoire diversity with clonal expansion across all patients, and this effect seems to be more prominent to group III of *DOCK8* mutation.

**Fig. 2:**
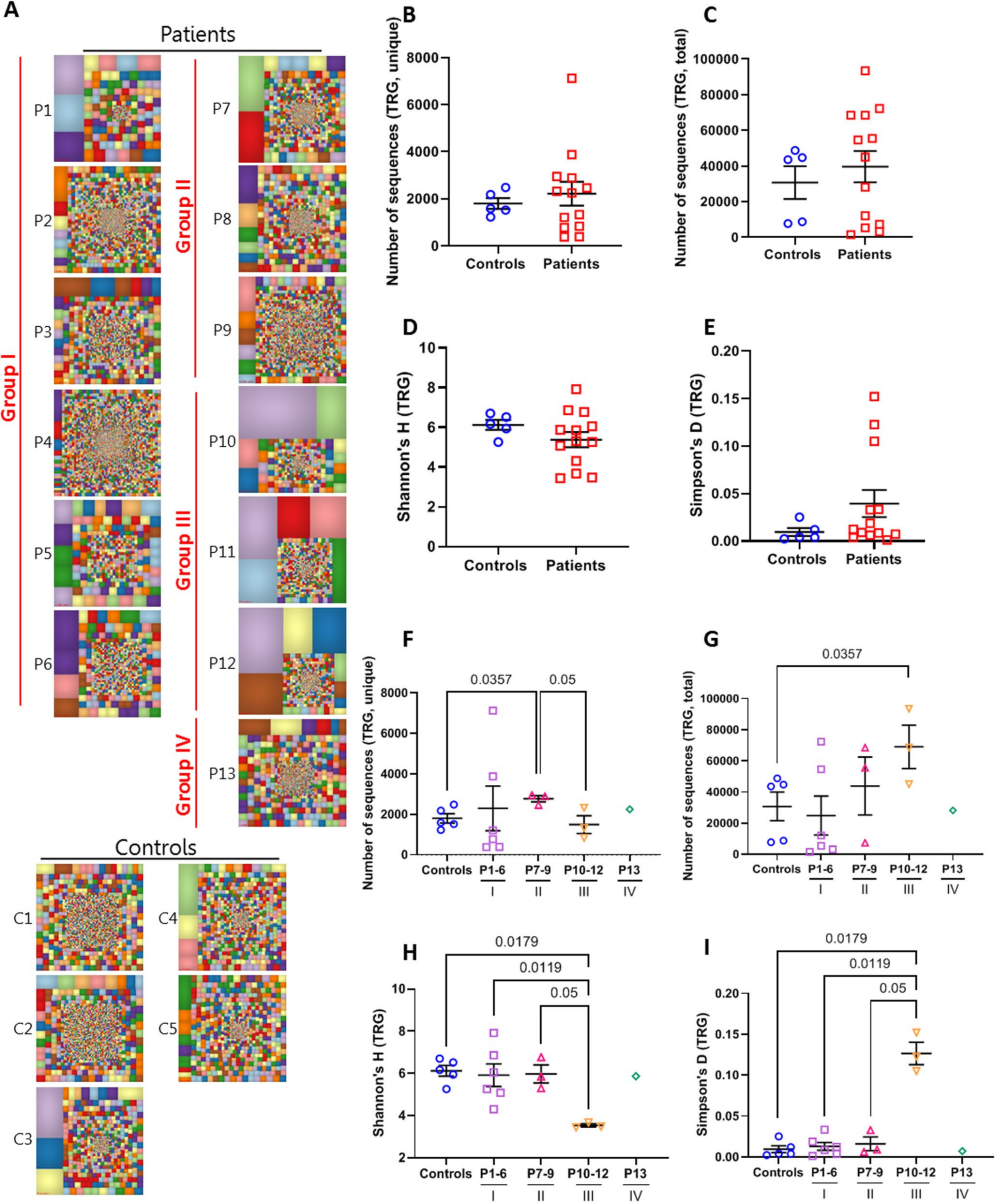
The T cell receptor γ (*TRG*) repertoire diversity in DOCK8 deficiency. (**A**) Treemap illustrations graphically and qualitatively present the diversity in *TRG* repertoire for patients and controls. Scatter dot plots for patients and controls presenting: (**B**) The unique number of sequences, which reflects the number of different clonotypes; (**C**) The number of total sequences, which portray the number of all circulating cells; (**D**) Shannon’s H index for diversity; and (**E**) Simpson’s D index of unevenness. Scatter dot plots comparing between controls and different groups of patients based on DOCK8 mutation type: (**F**) The unique number of sequences; (**G**) The number of total sequences; (**H**) Shannon’s H index for diversity; and (**I**) Simpson’s D index of unevenness. The whiskers in the graphs (**B-I**) present standard error (± SE). Statistics (**B-I)** performed using the Mann-Whitney U-test

Similarly, the *TRG* repertoire of DOCK8 deficient patients showed noticeable clonal expansions, as evident in the Treemaps (Fig. 2A). The unique and total number of sequences, the Shannon’s H diversity and Simpson’s indexes were distributed over a wide range in the patients compared to the controls (Fig. 2, B-E). Unlike *TRB* repertoire, Simpson’s D index was not significantly different in the *TRG* repertoire of the patients with DOCK8 deficiency compared to controls (Fig. 2E). When we analyzed the data according to different *DOCK8* mutations, the *TRG* repertoire of the patients in group II showed significantly higher number of unique sequences compared to controls and patients in group III (Fig. 2F). Patients in group III had a significantly higher total number of sequences compared to controls (Fig. 2G). Furthermore, patients from group III showed a significantly lower Shannon’s H diversity index with a significantly higher Simpson’s index of unevenness in the *TRG* repertoire compared to controls and patients from group I and II (Fig. 2, H and I). Thus, the *DOCK8* mutation of group III patients showed the most specific and robust effect, resulting in lower diversity of *TRG* repertoire with clonal expansion.

**Fig. 3:**
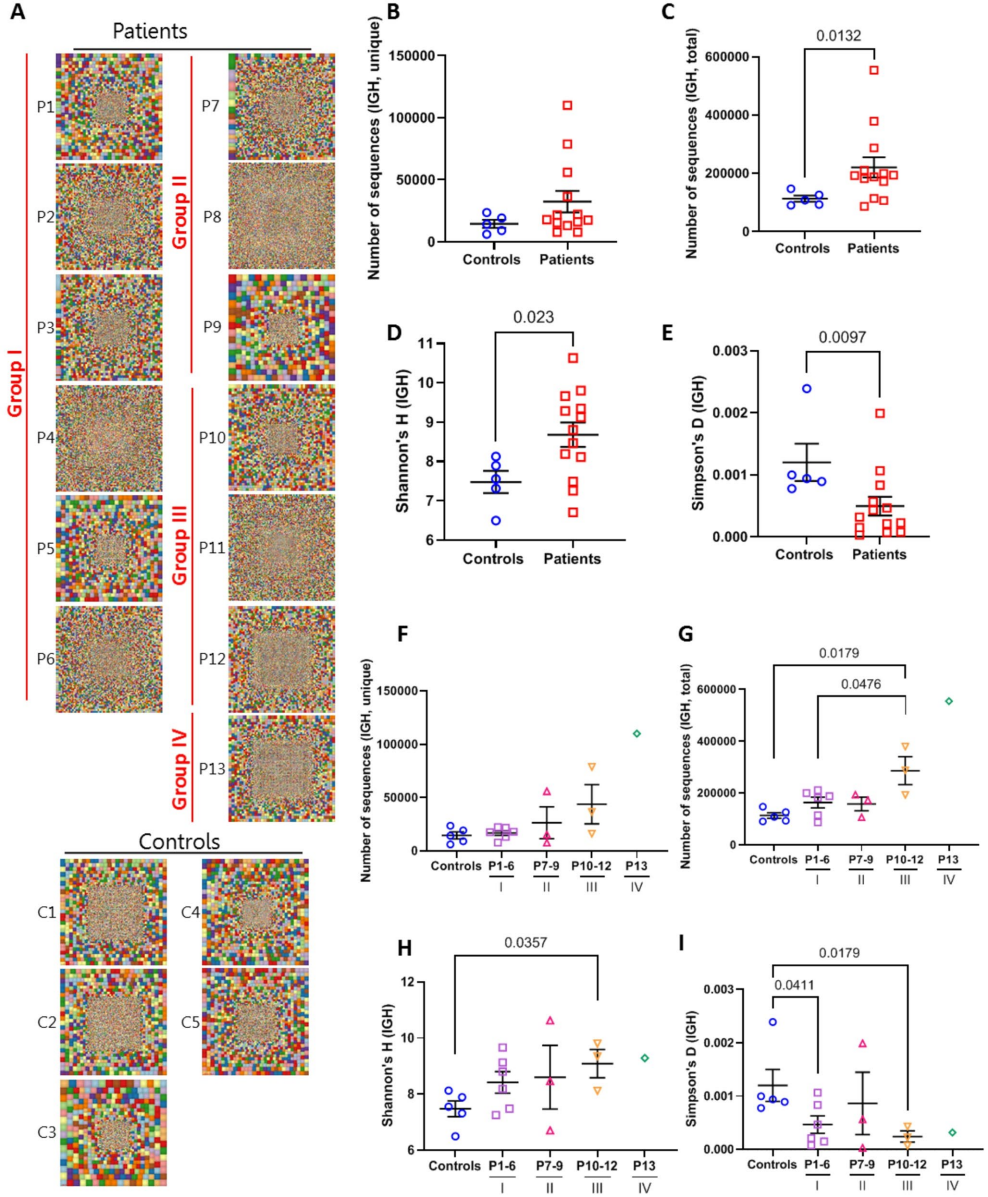
The Immunoglobulin heavy chain (*IGH*) of B cell receptor repertoire diversity in DOCK8 deficiency.(**A**) Treemap illustrations graphically and qualitatively present the diversity in *IGH* repertoire for patients and controls. Scatter dot plots for patients and controls presenting: (**B**) The unique number of sequences, which reflects the number of different clonotypes; (**C**) The number of total sequences, which portray the number of all circulating cells; (**D**) Shannon’s H index for diversity; and (**E**) Simpson’s D index of unevenness. Scatter dot plots comparing between controls and different groups of patients based on DOCK8 mutation type: (**F**) The unique number of sequences; (**G**) The number of total sequences; (**H**) Shannon’s H index for diversity; and (**I**) Simpson’s D index of unevenness. The whiskers in the graphs (**B-I**) present standard error (± SE). Statistics (**B-I**) performed using the Mann-Whitney U-test

In contrast to TCR repertoires, the Treemaps of *IGH* repertoire from the peripheral B-cells of DOCK8 deficient patients show slightly denser repertoire, with smaller squares compared to controls (Fig. 3A). Indeed, the number of both unique (Fig. 3B) and total (Fig. 3C) sequences of DOCK8 deficient patients’ *IGH* repertoire was high and significantly high (respectively) compared to controls, which corresponds to the normal to high levels of circulating B-cells present in these patients (Table 1). In addition, significantly high Shannon’s H diversity index (Fig. 3D) and significantly low Simpson’s D index (Fig. 3E) of the *IGH* repertoire were observed in patients compared to controls, indicative of a diverse and even repertoire. When we analyzed according to different *DOCK8* mutations (Fig. 3, F-I), group I showed significantly lower total number of sequences compared to group III (Fig. 3G) and significantly lower Simpson’s D index compared to controls (Fig. 3I). Group III showed significantly higher number of total sequences (Fig. 3G) and Shannon’s H diversity index (Fig. 3H), with significantly lower Simpson’s D index of unevenness (Fig. 3I). Hence, *DOCK8* mutations of group III had the most robust effect on the diversity of *IGH* repertoire.

To directly link these repertoire alterations to cellular function, we performed Spearman correlations between activation and developmental parameters (Table 1) and repertoire metrics within the DOCK8 deficient patient cohort (Table S1 and S2). PHA induced proliferation ratio positively correlated with unique number of TRB sequences (*r* = 0.9286, *p* = 0.0067, Table S3), indicating that preserved T cell activation capacity is associated with proper development of T cells. In contrast, no significant correlations were identified between B cell parameters and IGH repertoire metrics (Table S3).

### *TRB* and *TRG* Repertoires are Affected by Clonal Expansions but with Different Dynamics

**Fig. 4:**
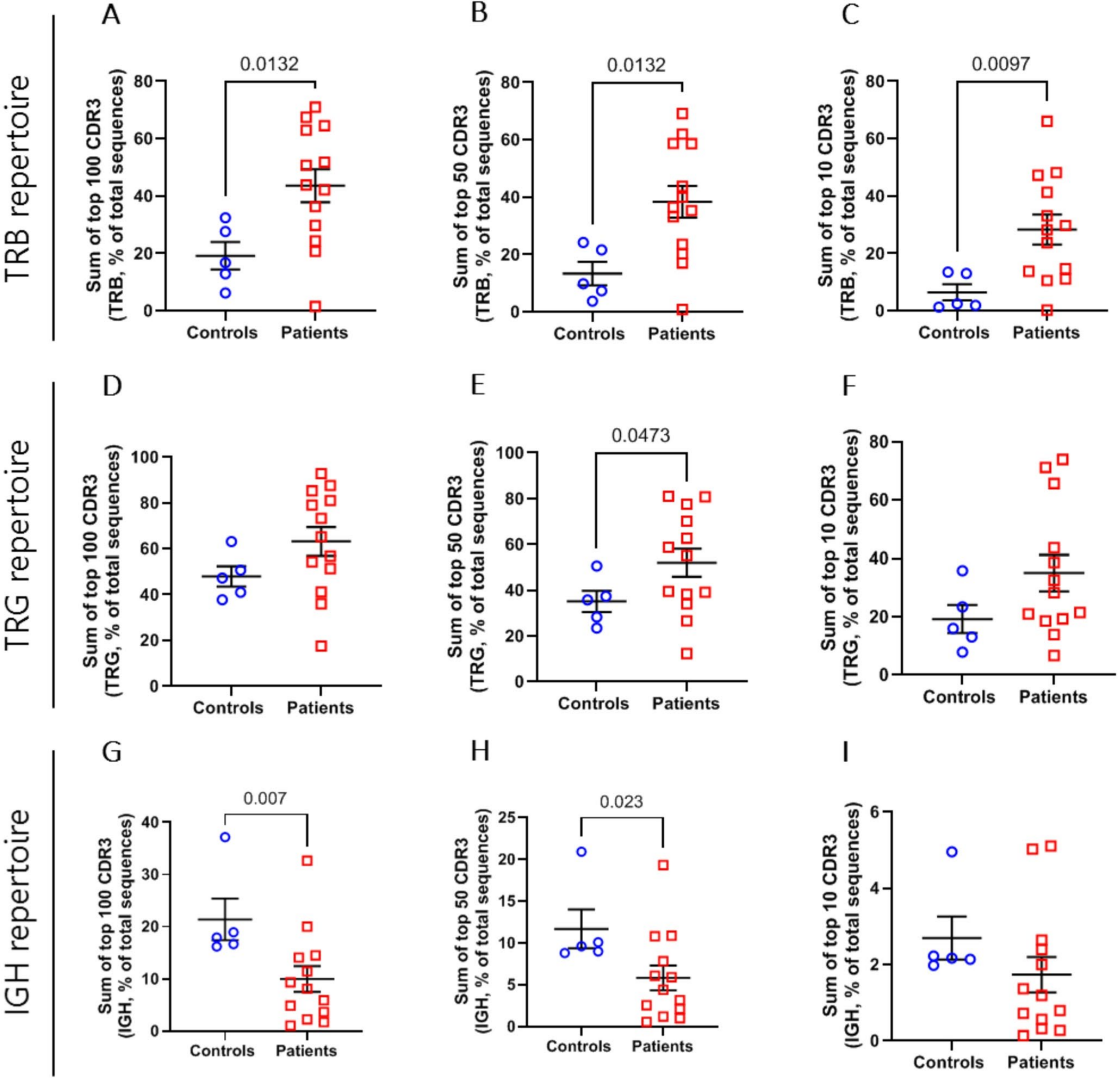
The dynamics of clonal expansions in T and B cells in DOCK8 deficiency. Scatter dot plots presenting the sum of top 100, top 50 and top 10 most abundant clones of patients’ and controls’ *TRB* (**A**-**C**), TRG (**D**-**F**) and IGH (**G**-**I**) repertoires. The whiskers in the graphs (**A-I**) present standard error (± SE). Statistics (**A-I**) performed using the Mann-Whitney U-test

In order to further elucidate the dynamics of clonal expansion, we analyzed the cumulative abundance of top 100, top 50 and top 10 most prevalent clones within the *TRB*, *TRG* and *IGH* repertoires (Fig. 4). In DOCK8 deficient patients, the TCR repertoire exhibited significantly higher percentages of clonal expansions compared to controls. Notably, the *TRB* repertoire featured larger clones among the top 10 clones than among the top 50 and top 100 clones (Fig. 4, A-C). In contrast, the *TRG* repertoire of these patients showed large clones consistently across the top 10, 50, 100 clones compared to controls, with top 50 showing a significantly larger clones in patients compared to controls (Fig. 4, D-F). The *IGH* repertoire in these patients showed significantly lower percentages of clonal expansions compared to controls, but only in the sum of top 100 and top 50 clones (Fig. 4, G-I).

To provide deeper insight into the composition of these expanded clones, Table S4 summarizes the V(D)J gene usages and CDR3 amino acid sequences of the 100 most abundant clones across DOCK8 deficient cohort. Additionally, Table 2 lists the TRB clonotypes shared among multiple patients (and were not shared with the top 100 clones in the controls) including the rank of the clone, *V*, *D* and *J* gene usage, and reported antigen specificities identified in public databases (VDJdb and McPAS-TCR). Several of these shared expanded clonotypes matched previously reported specificities for common pathogens, including cytomegalovirus (CMV), influenzaA and Epstein-Barr virus (EBV) (Table 2). Notably, patients harboring prominent clonal expansions had documented histories of recurrent cutaneous viral and bacterial infections (Table 1), which are hallmark complications of DOCK8 deficiency. Collectively, these data demonstrate the feasibility of identifying both pathogen associated and potentially DOCK8 deficiency specific clonotypes through high resolution TCR repertoire analysis.

**Table 2:** Shared CDR3 in the *TRB* repertoire in the top 100 most abundant clones

	CDR3 (AA)	CDR3 length (AA)	Clone number*	Individual	TRBV gene	TRBD gene	TRBJ gene	Copies	VDJdb	McPAS-TCR
1	ASSSLAGGPYEQY	13	1	P6	TRBV13	TRBD2	TRBJ2-7	44,798	CMV	
			1	P10	TRBV13	TRBD2	TRBJ2-7	11,514		
			6	P11	TRBV13	TRBD2	TRBJ2-7	697		
			24	P7	TRBV13	TRBD2	TRBJ2-7	177		
			38	P1	TRBV13	TRBD2	TRBJ2-7	61		
			60	P3	TRBV13	TRBD2	TRBJ2-7	37		
2	ASSYFSGRANNEQF	14	2	P6	TRBV6-5	TRBD2	TRBJ2-1	3,284		
			16	P10	TRBV6-5	TRBD2	TRBJ2-1	339		
			51	P11	TRBV6-5	TRBD2	TRBJ2-1	34		
3	ASSEWGEREQF	11	3	P6	TRBV25-1	TRBD2	TRBJ2-1	1,453		
			15	P10	TRBV25-1	TRBD2	TRBJ2-1	426		
4	ASSLWPPYEQY	11	4	P6	TRBV13	TRBD1	TRBJ2-7	787		
			22	P10	TRBV13	TRBD1	TRBJ2-7	246		
5	ASSQDIVGRGSLFYNSPLH	19	5	P6	TRBV4-3	TRBD2	TRBJ1-6	722		
			17	P10	TRBV4-3	TRBD2	TRBJ1-6	329		
6	ASSLGLAGGVSYNEQF	16	6	P6	TRBV7-8	TRBD2	TRBJ2-1	712		
			28	P10	TRBV7-8	TRBD2	TRBJ2-1	180		
			70	P11	TRBV7-8	TRBD2	TRBJ2-1	30		
7	ASSYSYEQY	9	7	P8	TRBV6-2		TRBJ2-7	186	InfluenzaA,CMV, EBV,	Cancer,autoimmune
			90	P13	TRBV6-2		TRBJ2-7	66		
8	ASSYGMWGGYT	11	7	P6	TRBV4-3	TRBD1	TRBJ1-2	523		
			20	P10	TRBV4-3	TRBD1	TRBJ1-2	257		
9	SASSWQTYNEQF	12	8	P6	TRBV20-1	TRBD1	TRBJ2-1	325		
			50	P10	TRBV20-1	TRBD1	TRBJ2-1	90		
10	ASSYNWDSNEKLF	13	9	P6	TRBV6-5	TRBD1	TRBJ1-4	291		
			79	P10	TRBV6-5	TRBD1	TRBJ1-4	53		
11	ASSREQGWETQY	12	11	P6	TRBV9	TRBD1	TRBJ2-5	179		
			100	P10	TRBV9	TRBD1	TRBJ2-5	40		
12	ASSQDLGVGTALLNTEAF	18	17	P6	TRBV4-3	TRBD1	TRBJ1-1	103		
			81	P10	TRBV4-3	TRBD1	TRBJ1-1	50		
13	SVDPYGQLNTEAF	13	19	P6	TRBV29-1	TRBD1	TRBJ1-1	74		
			78	P10	TRBV29-1	TRBD1	TRBJ1-1	54		
14	ASSPPSDSYEQY	12	21	P6	TRBV9	TRBD1	TRBJ2-7	71		
			88	P10	TRBV9	TRBD1	TRBJ2-7	45		
15	ATSRLGTGTAGYT	13	66	P6	TRBV15	TRBD1	TRBJ1-2	31		
			73	P11	TRBV15	TRBD1	TRBJ1-2	30		
16	ASSPALNTEAF	11	68	P13	TRBV18		TRBJ1-1	77		
			77	P12	TRBV18		TRBJ1-1	34		
17	ASSQDLAGGLLSYEQY	16	83	P10	TRBV4-3	TRBD2	TRBJ2-7	40		CMV
			32	P6	TRBV4-3	TRBD2	TRBJ2-7	43		

*clone number: from most abundant (1) to least abundant (100)

### Differential Gene Usage is Observed in the *TRG* Repertoire, but not in the *TRB* or *IGH* Repertoires, of Patients with DOCK8 Deficiency

**Fig. 5:**
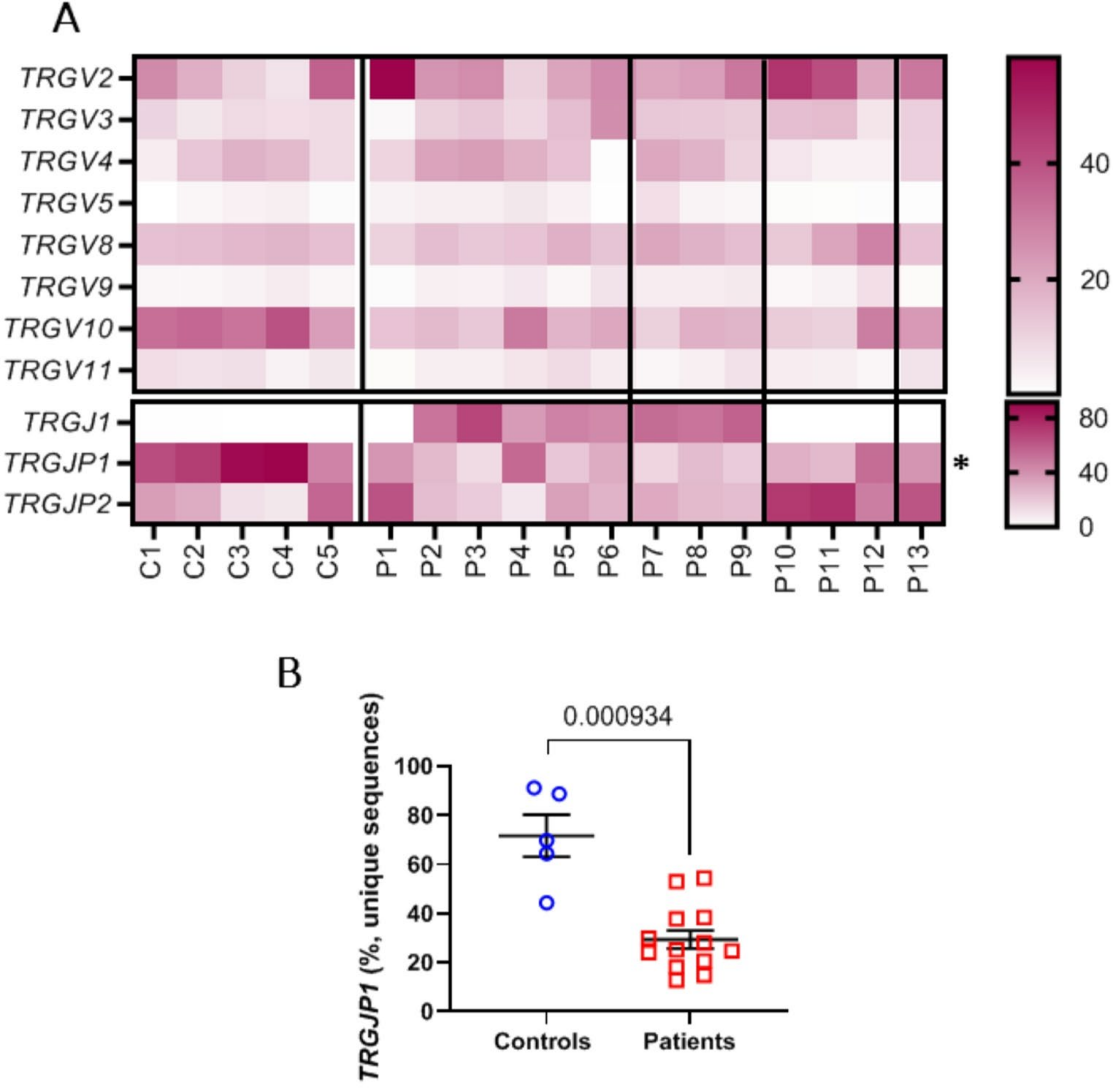
Differential ***J*** gene usages of *TRG* repertoire for unique sequences in DOCK8 deficiency. **A**. Heatmaps presenting percentages of *TRGV* and *TRGJ* gene usages in the unique sequences of the *TRG* repertoire for patients and controls. Scatter dot blot presenting the percentage of unique sequences that utilize *TRGJP1* (**B**) gene. The whiskers in the graphs (**B**) present standard error (± SE). Statistics using Multiple Mann-Whitney test with Benjammini, Krieger and Yekutieli method was applied to correct for multiple comparisons. Asterisk in the heatmaps (**A**) present specific gene usages that showed statistical significance

Since proper joining of the various *V*, *D* and *J* gene segments is crucial in generating diversity and functional immune repertoire, we analyzed the percentages of *V*, *D* and *J* gene usages of the *TRB*, *TRG* and *IGH* repertoire in DOCK8 deficient patients and compared them to healthy controls, both in unique and total sequences datasets. The comparison of the averages of each of the *TRBV*, *TRBD* and *TRBJ* gene usages in DOCK8 deficient patients showed no significant preferential gene usages compared to controls (Figure S2). Next, we examined the *TRGV* and *TRGJ* gene usages of the *TRG* repertoire in DOCK8 deficient patients and compared them to controls (Fig. 5A and Figure S3A). For both unique and total sequences, we found significantly deceased use of *TRGJP1* in DOCK8 deficient patients compared to controls (Fig. 5B and Figure S3B). These findings suggest potential alterations in the T cell receptor gamma repertoire that may be relevant to the immune dysregulation observed in DOCK8 deficiency. Finally, we analyzed the *IGHV*, *IGHD* and *IGHJ* gene usages of the *IGH* repertoire in DOCK8 deficient patients and controls, finding no significant differences in gene usages between the two groups (Figure S4, A and B). This suggests that the heavy chain repertoire remains relatively unaffected in DOCK8 deficiency.

### The Antigen Binding Region of the *TRB* Repertoire is Shifted Towards Longer CDR3 Lengths in Patients with DOCK8 Deficiency

**Fig. 6:**
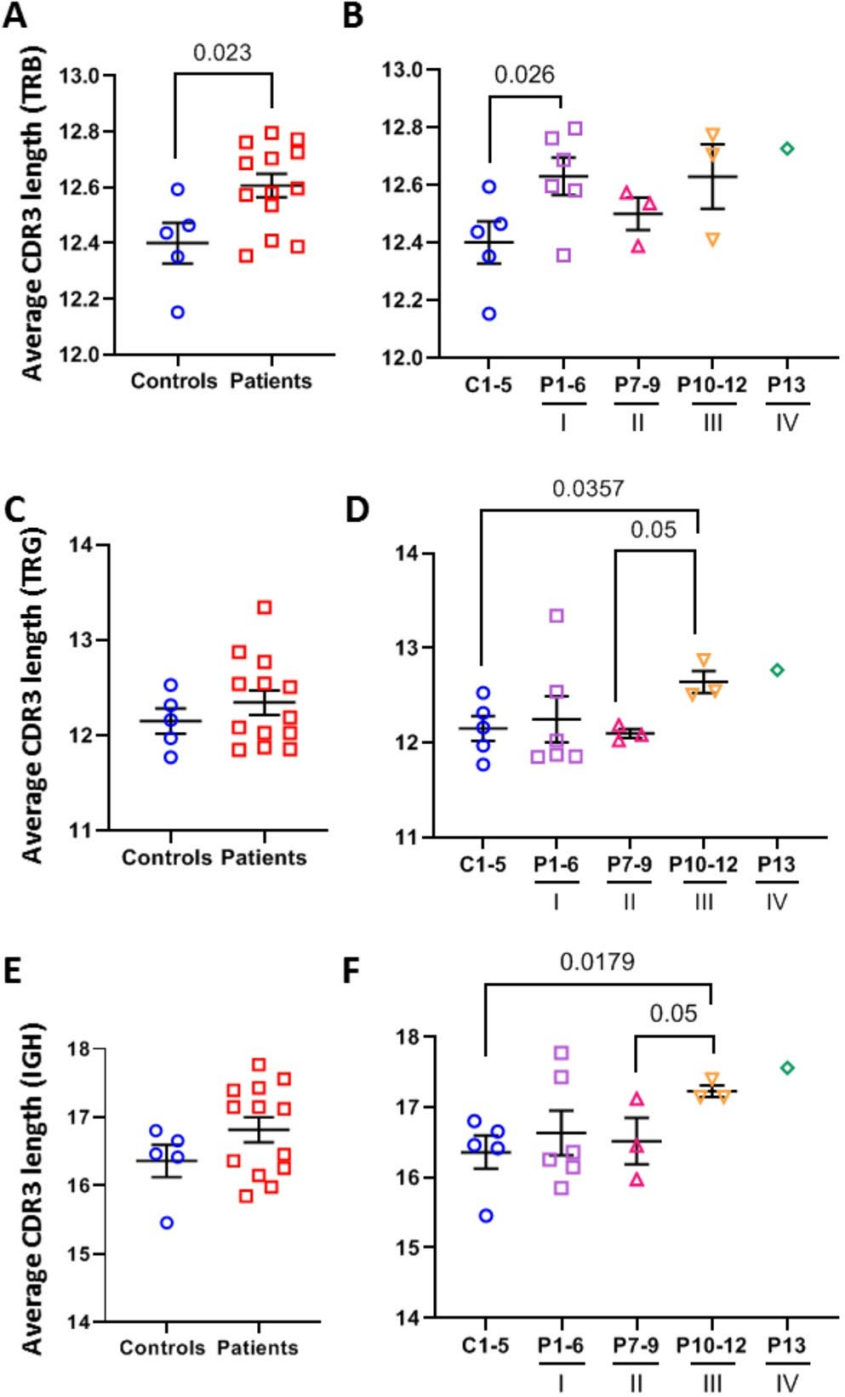
CDR3 lengths of *TRB*, *TRG* and *IGH* repertoire for unique sequences in patients with DOCK8 deficiency. Scatter dot plots for patients and controls presenting the average CDR3 lengths in unique sequences for *TRB*, *TRG* and *IGH*, for all the DIDS patients (**A**, **C**, and **E**, respectively) and according to DOCK8 mutation type (**B**,** D**, and **F**, respectively). The whiskers in the graphs (**A-F**) present standard error (± SE). Statistics (**A-F**) performed using the Mann-Whitney U-test. The reported CDR3 amino acid lengths in this study are two residues shorter than those typically obtained with tools that include the conserved amino acids in the CDR3 sequence as described in Material and Methods

An integral part of adaptive immune repertoire analysis is the characterization of the antigen binding site, known as the CDR3 region. Therefore, we analyzed the average CDR3 lengths for both unique and total sequences of DOCK8 deficient patients, and compared them to controls. In the *TRB* repertoire, the average CDR3 length of DIDS patients was significantly longer for both unique and total sequences compared to controls (Fig. 6A and Figure S5A). This distribution aligns with recent high-throughput NGS studies of healthy pediatric and adult cohorts with no significant age related differences [51]. The differences in average CDR3 length among the patients seem to segregate between groups I, III and IV on one side with longer CDR3 lengths in majority of the patients and patients in group II on the other side with comparable CDR3 lengths to controls, both in unique number of sequences (Fig. 6B) and total number of sequences (Figure S5B). Next, in the TRG repertoire, the average CDR3 length of the patients did not show any significant difference compared to controls, for both unique (Fig. 6C) and total sequences (Figure S5C). However, patients from group III showed a significantly longer CDR3 length compared to both controls and group II for the unique number of sequences (Fig. 6D) but not for the total number of sequences (Figure S5D). Lastly, similar to the *TRG* repertoire, in the *IGH* repertoire, the average CDR3 length of the patients did not show any significant difference compared to controls in unique sequences (Fig. 6E) but showed a significantly loner CDR3 in total (Figure S5E) sequences. Furthermore, patients from group III showed a significantly longer CDR3 length compared to controls and group II for both unique (Fig. 6F) and total (Figure S5F) number of sequences. Thus, *TRB* showed CDR3 length shifted towards longer lengths in patients with DOCK8 deficiency, where patients of group III consistently showed longer CDR3 length for *TRB*, *TRG* and *IGH* repertoire.

In order to further investigate the molecular mechanism for the significantly longer CDR3 of the *TRB* repertoire in the patients with DOCK8 deficiency, we analyzed to see whether there is an increase in nucleotide additions and/or decrease in trimming in the CDR3 region. Indeed, both the percentage of sequences with N nucleotide additions (Figure S6A) and average N nucleotide additions (Figure S6B) for the unique sequences of patients with DOCK8 deficiency showed a significantly higher values compared to controls. Furthermore, although the percentage of sequences with trimming were slightly higher in the patients with DOCK8 deficiency compared to controls (Figure S6C), the average number of nucleotides trimmed were comparable in both groups (Figure S6D). These analyses demonstrate an increased nucleotide additions in the *TRB* repertoire contribute to the generation of longer average CDR3 length observed in patients with DOCK8 deficiency. Potential mechanisms include altered thymic selection and post thymic survival bias, however, the functional relevance of this bias requires further investigation.

### The Maturation of the *IGH* Repertoire may be Affected in the Patients with DOCK8 Deficiency

Somatic hypermutations are a key indicator of B-cell maturity, occurring during the late stages of B-cell differentiation after mature naïve B-cells are activated. In our study, we used the pid score from IMGT HighV-QUEST to quantify the similarity of the *IGH* repertoire to germline sequences. A lower pid score indicates a higher level of somatic hypermutations. Our results showed that patients with DOCK8 deficiency had a higher average pid score compared to controls for both unique (Figure S7A) and total (Figure S7B) sequences, although this difference was not statistically significant.

## Discussion

This study involves a comprehensive clinical and immunological evaluation of 13 patients diagnosed with DOCK8 deficiency. Since DOCK8 is a regulator of the actin cytoskeleton and formation of immunological synapses, we speculated that DOCK8 deficient patients will display abnormal TCR and BCR repertoires. Therefore, we utilized NGS technologies to characterize the molecular signatures of DOCK8 deficient patients’ immune repertoire from peripheral blood. Notably, we showed unique abnormalities in the TCR and BCR repertoires of patients with DOCK8 deficiency, which correspond to the clinical and immunological data of the patients and *DOCK8* mutations. In the TCR repertoire, as summarized in Table 3, DOCK8 deficient patients showed a pronounced uneven repertoire for both *TRB* and *TRG* due to clonal expansions (Simpson’s D). Interestingly, the dynamics of clonal expansions are different between *TRB* and *TRG* in DOCK8 deficient patients; a substantial number of circulating αβ T-cells are derived from the most abundant 10 clonotypes, which is not the case with γδ T-cells. Taken together, TCR repertoire suggests that mutated DOCK8 impacts the activation and proliferation of T cells in the periphery rather than in the initial stages of T-cell development. The positive correlation between PHA responsiveness and unique *TRB* sequences within the DOCK8 patient cohort provides a direct functional link between degree of T cell activation impairment and the extent of TRB repertoire skewing.

**Table 3:** Summary of immune repertoire parameters that showed significant differences compared to controls, for all patients and for the three patient groups with specific DOCK8 mutations. Unique-Unique number of sequences; Total - Total number of sequences; CDR3-Unique - CDR3 length for the unique sequence dataset; CDR3-Total - CDR3 length for the total sequence dataset

	Unique	Total	Shannon’s H	Simpson’s D	CDR3-Unique	CDR3-Total
All Patients						
TRB				High	Long	Long
TRG						
IGH		High	High	Low		Long
Group I						
TRB					Long	Long
TRG						
IGH				Low		
Group II						
TRB						
TRG	High					
IGH						
Group III						
TRB			Low	High		
TRG		High	Low	High	Long	
IGH		High	High	Low	Long	Long

Discrepancies between TCR-Vβ flow cytometry and NGS based TRB repertoire sequencing reflect fundamental differences in resolution. In Patient 12, for example, flow cytometry indicated an oligoclonal repertoire, whereas NGS revealed a diversity comparable to healthy donors. TCR-Vβ flow cytometry provides a macroscopic view of Vβ family usage at the protein level, whereas NGS resolves individual CDR3 clonotypes generated by *V(D)J* rearrangement, junctional diversity, and N-nucleotide addition. Consequently, apparent oligoclonality detected by flow cytometry may mask substantial underlying clonal diversity at the nucleotide level. NGS therefore provides a more precise assessment of true repertoire diversity and clonality, while TCR-Vβ flow cytometry remains a rapid, clinically accessible tool.

Detailed characterization of expanded *TRB* clones in DOCK8 deficient patients reveals both shared and private features of the TCR repertoire. Identification of several public clonotypes matching known specificities for CMV, influenzaA and EBV, combined with clinical history of recurrent cutaneous viral and bacterial infections, indicates that a substantial fraction of these expansions represent antigen driven responses to pathogens to which DOCK8 deficient individuals are highly susceptible. Importantly, additional shared clonotypes absent from public TCR databases and from most abundant clonotypes of healthy controls suggest that TCR repertoire sequencing may enable the discovery of clonotypes specifically associated with DOCK8 deficiency. Such clonotypes may reflect dysregulated responses to self-antigens, altered selection thresholds in the absence of functional DOCK8, or reactivity to uncharacterized microbial antigen that preferentially drive expansion in this genetic background. However, because antigen specific TCR sequencing, single cell functional assays, or longitudinal sampling were not performed, we cannot definitely attribute individual CDR3 sequences to particular clinical events or ongoing infections and need further investigations.

Several lines of evidence indicate that the observed TRB repertoire alterations originate early during T cell development in DOCK8 mutant progenitors. First, the characteristic lengthening of TRB-CDR3 region was consistently detected in both unique and total sequence dataset, arguing against a primary contribution from antigen driven clonal expansions in the periphery. Second, DOCK8 deficient patients showed significantly increased N-nucleotide additions in TRB repertoire in both unique and total sequence datasets. Third, the preferential usage of the longer TRBD2 gene in 7 out of 13 patients in our patient cohort and as was previously shown [30]. These junctional changes argue against a purely peripheral and antigen driven, but instead support a cell intrinsic defect at the level of *V(D)J* recombination and/or early thymic selection, however, direct confirmation in sorted progenitor cells awaits future studies.

Abnormalities of the CDR3 region often affect the ability to create proper immune responses, specifically to self-antigens leading to autoimmune manifestations [31], a leading characteristic of DOCK8 deficiency. Thereby, longer CDR3 length in αβ T-cells may contribute to the mechanism leading to autoimmune phenotype in DOCK8 deficiency. In addition, *TRB* repertoires in healthy adults were associated with shorter CDR3 [52], specifically in memory T cells. Furthermore, TCR sequencing of αβ T-cells revealed repertoire characteristics of self-reactive CD8 + T-cell clones in DOCK8 deficiency [42]. It is noteworthy that Bozkurt et al. [42] used a different methodology of using RNA from sorted CD4 + and CD8 + T cells to sequence the V-D-J rearrangements, while we used genomic DNA from unenriched whole blood samples. Our genomic DNA approach provides a broader, less biased profile of lymphocyte clonotypes proportional to cell numbers, whereas the RNA method offers higher sensitivity for expressed, functional TCRs but biased towards activated/expanded clones. Nonetheless, Bozkurt et al. [42] linked elevated cysteine/hydrophobic CDR3 motifs in DOCK8 deficient CD8 + TRB to self-reactivity and non-canonical differentiation. Altogether, these findings correspond to our data that mutations in *DOCK8* may lead to the generation of pathological and autoreactive αβ T-cell development, due to defects during junctional diversity and *V-D-J* rearrangement process.

Preferential gene usage is another parameter which can associate with specific disease, as was observed with *IGHV4-34* and lupus [53]. In DOCK8 deficient patients, only *TRG* repertoire showed reduced utilization of *TRGJP1*. Nonetheless, while patients who did not use *TRGJ1*, did not utilize *TRGJP1* to the same extent as controls, they instead favored *TRGJP2*. This implies that DOCK8 plays a role in an initial molecular process, given that these changes were evident in both unique and total sequences. We speculate that our findings are in line with DOCK8 studies and “actinopathies”, strengthening the possible connections between DOCK8 and molecular mechanisms of genomic accessibility [34, 36, 54, 55] and regulation of *VDJ* recombination process. 

The *IGH* repertoire analysis of DOCK8 deficient patients demonstrated notable increase in peripheral blood B-cells compared to controls, which correlates with clinical features of normal to high B-cell counts with elevated serum immunoglobulins concentrations and defective antibody response observed in our DOCK8 deficient patients’ cohort. Furthermore, the unexpectedly high diversity attributed to abundance of clonotypes without clonal expansions. Thus, *IGH* repertoire can possibly depict the defects in immunological synapse formation leading to abnormal selection processes in B-cell development [56], allowing for increased possibilities of unwanted reactivity. Thereby, DOCK8 deficiency may lead to break in central tolerance, implying for a molecular mechanism for atopy and autoimmunity [57]. However, further investigation is needed to directly link these highly diverse *IGH* repertoire with unwanted reactivity such as atopy and autoimmunity. In addition, although the somatic hyper mutation is preserved, the lack of clonal expansion suggests inadequate activation and proliferation of B-cells in DOCK8 deficiency.

We have previously reported immune repertoire studies in other IEIs such as WAS and ARPC1B deficiency, with a similar pathomechanism as DOCK8 deficiency involving cytoskeleton regulation [37, 40]. Since skin disorders are known to be associated with γδ T-cells in the periphery, studying the *TRG* repertoire characteristics in diseases with eczematous phenotype is important. Indeed, abnormalities in the *TRG* repertoire were observed in these IEIs, but each actinopathy displayed a unique feature which can contribute to specific pathomechanism in the overall function of γδ T-cells. In DOCK8 deficiency, substantial clonal expansion and preferential gene usages in the *TRG* repertoire was observed while WAS patients showed low number of total sequences [37] and ARPC1B deficient patients presented with preferential usages of *TRGV4* gene [40]. It is noteworthy that the *IGH* repertoire of these IEIs share similar dynamics of over-diverse repertoire without clonal expansion, implying a common inadequate activation in B-cells in these disorders. However, what distinguishes the present DOCK8 study is the substantially larger patient cohort, which enables us to resolve genotype repertoire correlations and to identify mutation specific effects on repertoire architecture, as was previously been demonstrated for RAG deficient patients [31]. These findings underscore that, while the overreaching repertoire phenotype is shared among cytoskeletal immunodeficiencies, DOCK8 deficiency additionally permits the detection of mutation driven, patient specific repertoire signatures.

Immune repertoire analysis according to different mutations in *DOCK8* revealed a possible mutation specific characteristics (Table 3), correlating to clinical presentation and immunological data. Based on previous studies, deletion mutations of group III are less likely to undergo spontaneous somatic repair, thereby leading to a more severe disease [16, 18, 19]. Indeed, group III is outstanding in revealing significant results of *TRG* and *IGH* repertoire parameters which were not observed when comparing to all the DOCK8 deficient patients together (Table 3). Specifically, reduced diversity, clonal expansion, high number of total sequences and longer CDR3 length of *TRG* repertoire suggest a pathological effect on γδ T-cell to associate with the severe mutations of group III. Furthermore, these severe mutations lead to longer CDR3 length of *IGH* repertoire without clonal expansions, complementing previous study that showed shorter *IGH* CDR3 in expanded clones compared to non-expanded clones [56]. Longer CDR3 is linked to autoimmune phenotype [58], and interestingly, all the patients in group III present with autoimmunity manifestations. Lastly, *TRB* diversity parameters of group III showed consistent results, unlike other groups. The mutation group I is characterized with more severe clinical course and manifestations [45], and together with group III showed higher number of total *TRB* and *IGH* sequences in addition to higher diversity without clonal expansion in *IGH* repertoire. To note, the mutations in groups I and III are all in the same DHR-2 domain, crucial for the GEF activity of DOCK8 protein, which is responsible for proper cell migration upon activation [5, 59]. Taken together, these analyses suggest interdependence between specific DOCK8 mutation, immune repertoire, and clinical manifestations alongside disease severity.

Although the findings of current study are informative, several limitations should be noted. First, somatic reversion was not assessed, so we could not determine whether partial DOCK8 rescue influenced the immune repertoire in any of the patients. Second, since for clarity the cohort is ultra-rare and spans a wide age range, we could not perform age stratified or age adjusted analyses, thus, age related confounding effects cannot be excluded. Third, this study is descriptive and associative, and does not establish causality between repertoire alterations and clinical manifestations. Future studies with DOCK8 protein analysis, mosaicism assessment, and longitudinal TCR and BCR profiling will be needed to validate and extend these findings.

Our study offers insights of how various different *DOCK8* mutations influence the adaptive immune repertoire via defining unique molecular signature, different from both controls and from IEIs with similar pathomechanism. Specifically, TCR repertoire revealed a possible impairment in T-cell development and activation of both αβ and γδ T-cells, and a suggestive mechanism for the autoimmune phenotype in the absence of DOCK8. In addition, BCR repertoire portrayed an abnormal B-cell development that may lead to a possible mechanism for atopy and autoimmunity, alongside inadequate activation in the periphery in DOCK8 deficiency. In conclusion, our study uniquely demonstrates that immune repertoire analysis can uncover mutation specific adaptive immune defects and help explain the distinct phenotypes associated with DOCK8 deficiency.

## Supplementary Information

Below is the link to the electronic supplementary material.

Supplementary Material 1

Supplementary Material 2 (XLSX 79.4 KB)

## Data Availability

All sequencing data generated in this study have been deposited in Figshare and are publicly available at https://doi.org/10.6084/m9.figshare.31996116
