# Supplementary material for: Comprehensive Profiling of T- and B-Cell Receptor Repertoires Demonstrates Impaired Developmental and Activation of Adaptive Immunity in DOCK8 Deficiency
Source: J Clin Immunol. 2026 May 6;46(1):69. doi: 10.1007/s10875-026-02031-z (PMC13314901; doi:10.1007/s10875-026-02031-z)
Supplement: Supplementary file 1 — Supplementary Material 1 [file 10875_2026_2031_MOESM1_ESM.docx]

# Supplementary Table, Figures and Figure Legend

**Table S1.** **Number of sequences for the *TRB*, *TRG* and *IGH* repertoires obtained from the peripheral blood samples of DOCK8 deficient patients and controls**

| **Group** | | **Age at sampling** | **TRB** | | **TRG** | | **IGH** | |
| --- | --- | --- | --- | --- | --- | --- | --- | --- |
|  |  |  | **Unique number of sequences** | **Total number of sequences** | **Unique number of sequences** | **Total number of sequences** | **Unique number of sequences** | **Total number of sequences** |
| Patients | P1 | 8 y | 1,976 | 30,978 | 386 | 12,140 | 13,224 | 187,812 |
|  | P2 | 5 mo | 6,434 | 62,504 | 3,879 | 54,666 | 17,902 | 181,655 |
|  | P3 | 4 y + 2 mo | 1,877 | 24,621 | 1,208 | 5,390 | 17,518 | 199,844 |
|  | P4 | 4 mo | 33,682 | 65,673 | 7,126 | 72,273 | 21,673 | 86,727 |
|  | P5 | 7 y + 2 mo | 2,070 | 22,264 | 393 | 1,517 | 7,855 | 114,001 |
|  | P6 | 2 y + 8 mo | 4,063 | 80,456 | 776 | 3,212 | 22,346 | 210,620 |
|  | P7 | 2 y | 16,043 | 62,888 | 2,953 | 68,516 | 15,481 | 193,593 |
|  | P8 | 2 y + 4 mo | 7,670 | 21,894 | 2,891 | 55,562 | 56,086 | 172,368 |
|  | P9 | NA | 2,116 | 24,740 | 2,464 | 7,393 | 7,848 | 106,939 |
|  | P10 | 8 y + 2 mo | 3,280 | 49,792 | 836 | 45,035 | 16,241 | 192,111 |
|  | P11 | 1 y + 11 mo | 5,864 | 47,496 | 1,331 | 68,529 | 36,545 | 287,308 |
|  | P12 | 7 y + 10 mo | 4,061 | 30,949 | 2,321 | 93,456 | 78,822 | 378,907 |
|  | P13 | 11 y + 6 mo | 7,559 | 59,562 | 2,251 | 28,201 | 110,119 | 554,191 |
| Controls | C1 | 3 y + 10 mo | 9,220 | 35,136 | 2,185 | 7,753 | 23,742 | 108,498 |
|  | C2 | 10 y + 7 mo | 3,866 | 32,924 | 2,480 | 8,783 | 19,312 | 90,643 |
|  | C3 | 1 mo | 3,153 | 23,222 | 1,229 | 44,784 | 6,132 | 93,329 |
|  | C4 | 1 mo | 3,867 | 24,018 | 1,591 | 48,771 | 9,191 | 126,372 |
|  | C5 | 1 y + 2 mo | 2,445 | 17,819 | 1,523 | 43,591 | 14,312 | 147,011 |
| *TRB* T-cell receptor β chain, *TRG* T-cell receptor γ chain, *IGH* immunoglobulin heavy-chain, *mo* month, *y* year, *ND* not done, *NA* not available | | | | | | | | |

**Table S2. Summary of Shannon’s H and Simpson’s D diversity indices for *TRG*, *TRG* and *IGH* repertoires obtained from peripheral blood samples of DOCK8 deficient patients and controls**

**Table S3. Summary of the Spearman’s rank correlation test**

Correlations were assessed in DOCK8 deficient patients.

**Table S4. List of the most abundant Top 100 TRB clones and V, D and J gene usage in DOCK8 deficient patients and healthy controls (Table S4 is in separate file)**

**Figure S1. Age at sampling of the DOCK8 deficient patients and controls.** Scatter dot plots for patients and controls presenting the age of the healthy control donors and patients at the time of blood sampling. The whiskers in the graphs present standard error (± SE). Statistics performed using the Mann-Whitney U-test.


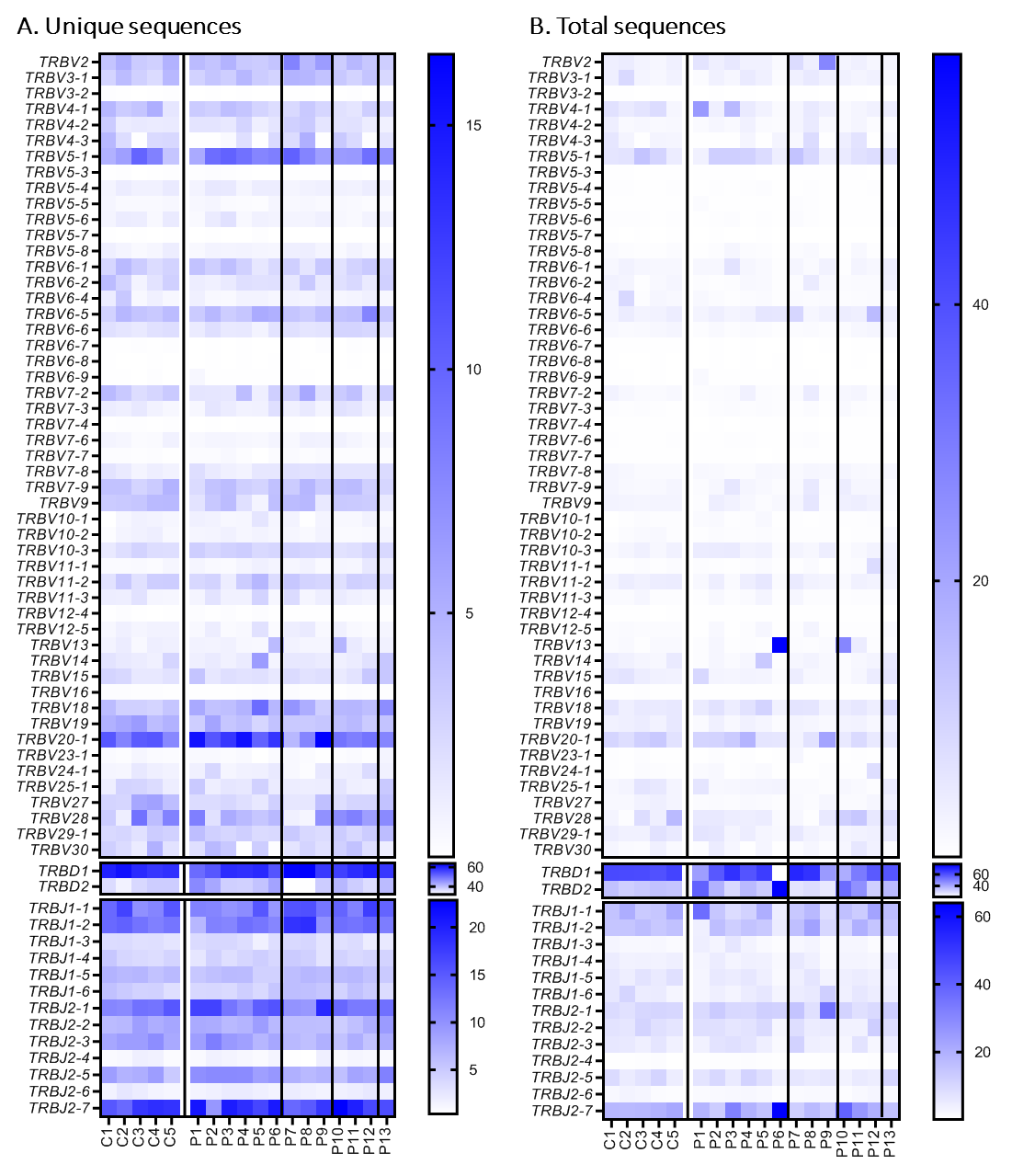


**Figure S2. The *V*, *D*, and *J* gene usages of *TRB* repertoire for unique and total sequences in DOCK8 deficiency**. Heatmaps for patients and controls presenting percentages of *TRBV*, *TRBD* and *TRBJ* gene usages of the *TRB* repertoire; for unique sequences (**A**), and total sequences (**B**). Statistics performed using multiple Mann-Whitney test with Benjammini, Krieger and Yekutieli method.


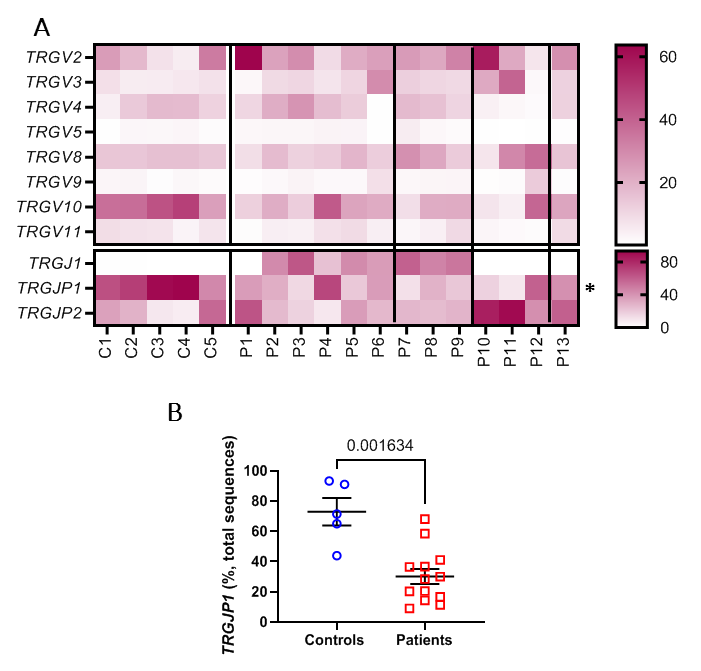


**Figure S3. Differential *J* gene usages of TRG repertoire for total sequences in DOCK8 deficiency**. **A**. Heatmaps presenting percentages of *TRGV* and *TRGJ* gene usages in the total sequences of the TRG repertoire for patients and controls. Scatter dot blot presenting the percentage of unique sequences that utilize *TRGJP1* (**B**) gene. The whiskers in the graph (**B**) present standard error (± SE). Statistics performed using the multiple Mann-Whitney U-test with Benjammini, Krieger and Yekutieli method. Asterisk in the heatmaps (**A**) present specific gene usages that showed statistical significance.

**
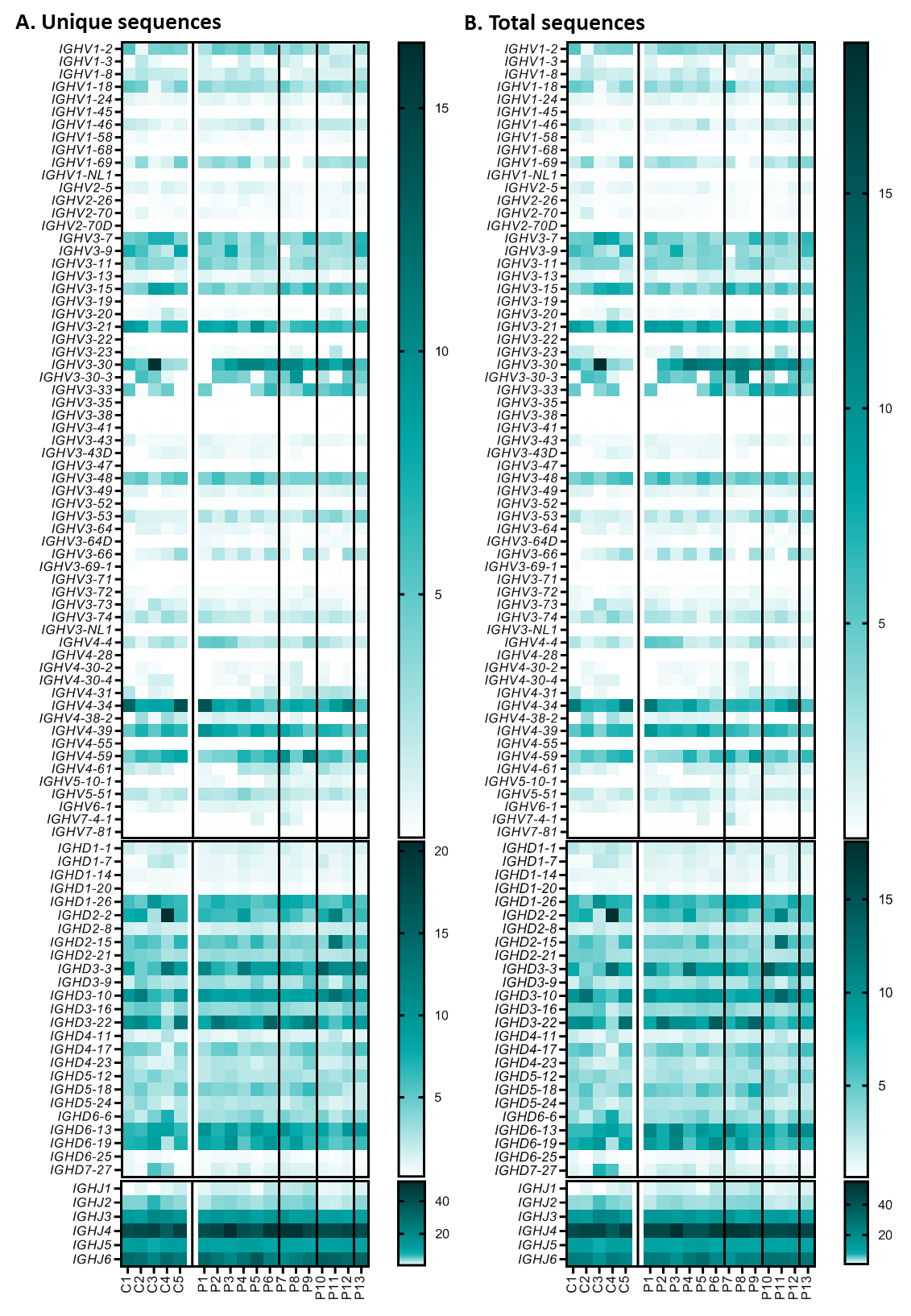
**

**Figure S4**. **The *V*, *D*, and *J* gene usages of *IGH* repertoire for unique and total sequences in DOCK8 deficiency.** Heatmaps for patients and controls presenting percentages of *IGHV*, *IGHD* and *IGHJ* gene usages of the TRB repertoire; for unique sequences (**A**), and total sequences (**B**). Statistics performed using multiple Mann-Whitney test with Benjammini, Krieger and Yekutieli method.

**
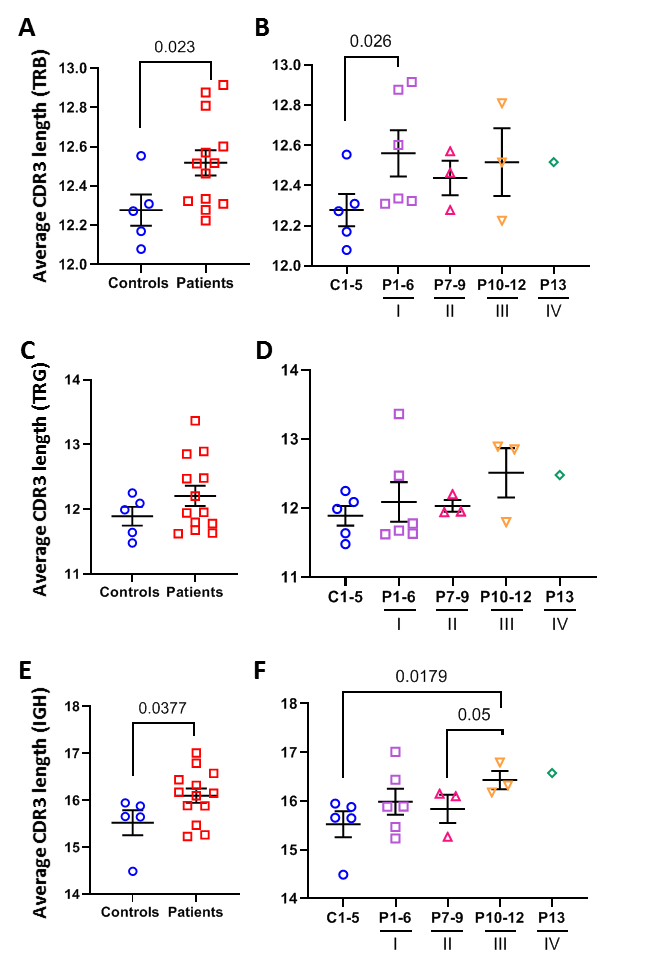
**

**Figure S5. CDR3 lengths of *TRB*, *TRG* and *IGH* repertoire for total sequences in patients with DOCK8 deficiency.** Scatter dot plots for patients and controls presenting the average CDR3 lengths in the total number of sequences for *TRB*, *TRG* and *IGH*, for all the DIDS patients (**A**, **C**, and **E**, respectively) and according to DOCK8 mutation type (**B, D,** and **F**, respectively). The whiskers in the graphs (**A-F**) present standard error (± SE). Statistics (**A-F**) performed using the unpaired Mann-Whitney U-test.

**
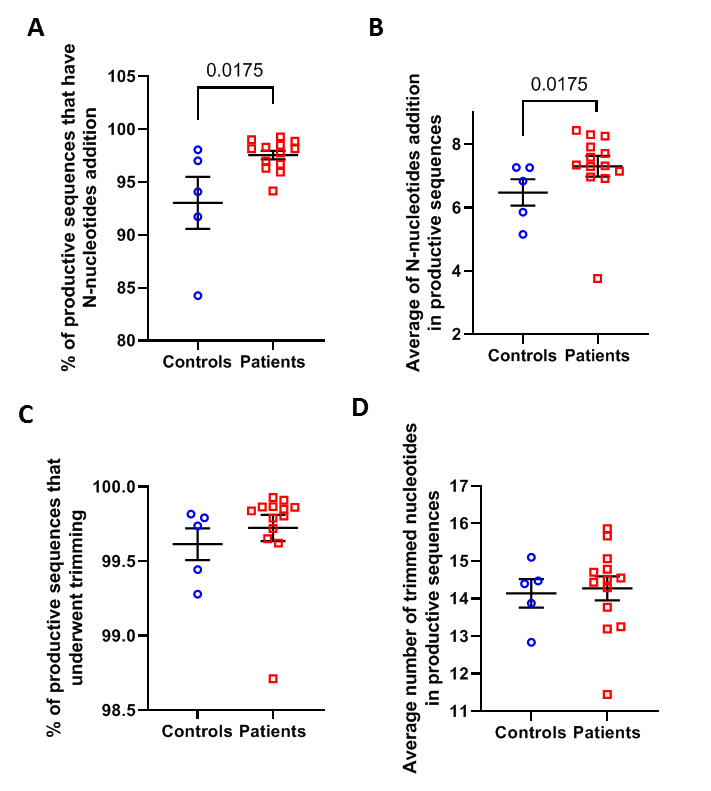
**

**Figure S6. Nucleotide addition and trimming in the *VDJ* junctions of the *TRB* repertoire in DOCK8 deficient patients.** Scatter dot plots for patients and controls describing nucleotides addition and deletion, which collectively create the junctional diversity in *V-D-J* junctions of *TRB* repertoire: **A**. The percentage of productive sequences that have N-nucleotides addition; **B**. The average number of N-nucleotides which have been added in productive sequences. **C**. The percentage of productive sequences that underwent trimming. **D**. The average number of trimmed nucleotides in productive sequences that underwent trimming. The whiskers in the graphs (**A-D**) present standard error (± SE). Statistics (**A-D**) performed using Mann-Whitney U-test.

**
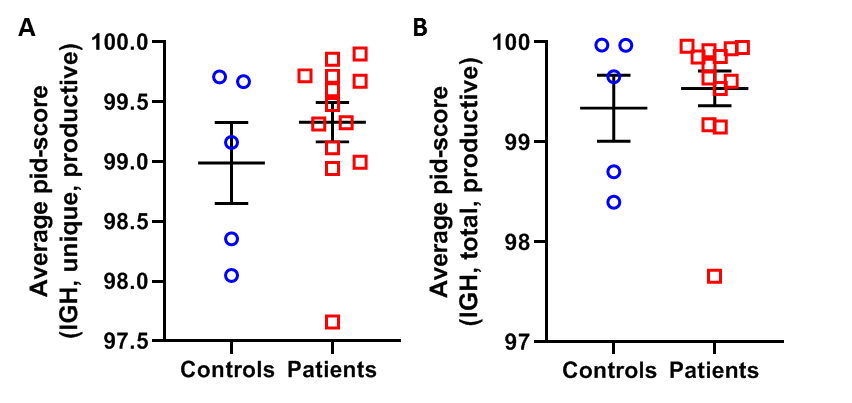
**

**Figure S7.** **Assessment for presence of somatic hypermutation in the *IGH* repertoire of DOCK8 deficient patients**. Scatter dot plots for patients and controls presenting the average pid-scores of *IGH* repertoire, which accounts for the percent identity with the germline *V* gene, in unique (**A**) and total sequences (**B**). Statistics (**A-D**) performed using the unpaired Mann-Whitney U-test.
